# Mycotic Aneurysm after Bacillus Calmette-Guérin Treatment: Case Report and Review of the Literature

**DOI:** 10.1155/2017/4508583

**Published:** 2017-02-20

**Authors:** Nathaniel D. Coddington, Jesse K. Sandberg, Chen Yang, Jennifer K. Sehn, Eric H. Kim, Seth A. Strope

**Affiliations:** Washington University School of Medicine in St. Louis, St. Louis, MO, USA

## Abstract

*Background*. Intravesicular Bacillus Calmette-Guérin (BCG) is an effective adjunctive therapy for superficial bladder cancer that has been shown to delay recurrence and progression of disease. Serious side effects are relatively rare but are difficult to diagnosis and commonly overlooked.* Case Presentation*. We report the case of a patient who was found to have mycotic aortic aneurysms secondary to treatment with BCG after a prolonged course with multiple intervening hospitalizations.* Conclusion*. Through this report, we discuss our present understanding of BCG infection following treatment and review the literature regarding this particular rare manifestation.

## 1. Background

Bacillus Calmette-Guérin (BCG) is a live attenuated strain of* Mycobacterium bovis* commonly used in the management of high-risk non-muscle-invasive bladder cancer [1]. Intravesicular instillation of BCG is believed to provoke an inflammatory response which when used as an adjunct to transurethral resection of visible tumor (TURBT) has been shown to delay both the recurrence rate and the progression of bladder cancer [2]. Side effects are generally tolerable; while up to 90% of patients experience cystitis and flu-like symptoms for 24 to 48 hours after treatment, multiple series have shown that serious infections occur in less than 5% of patients overall [3, 4]. These infections are usually either limited to local structures within the genitourinary tract or disseminated with fever and organ involvement. Mycotic aneurysm is a rare and often fatal presentation of this syndrome [5].

We report here a case of a 72-year-old man who was diagnosed with a mycotic aneurysm secondary to BCG 8 months after his most recent treatment.

## 2. Case Presentation

The patient is a 72-year-old man with a history of bladder cancer diagnosed two years ago now* status post* two rounds of intravesicular BCG. His most recent treatment was given 8 months prior to his ultimate diagnosis with BCG-positive mycotic aneurysms. The indication for treatment was a recurrence in carcinoma in situ that was picked up on routine screening. There was no trauma or concern for infection noted at that time.

He initially presented to an outside emergency room one month after this second round of BCG with recurrent fevers and rigors. Urine cultures grew coagulase-negative* Staphylococcus aureus* and he was sent home on Macrobid. He then presented shortly thereafter with increasing lethargy and was transferred to our facility where he was found to have an acute kidney injury and pancytopenia of unclear etiology. His AKI resolved with hydration and work-up for his pancytopenia was notable for a thyroid nodule that was biopsied but ultimately found to be benign. Imaging at that time was notable for a normal abdominal aorta. The patient declined a bone marrow biopsy. He would then present once more a few months later to an outside facility with worsening rigors, chills, and fevers. A chest X-ray was concerning for pneumonia which was treated with cefuroxime.

**Figure 1:**
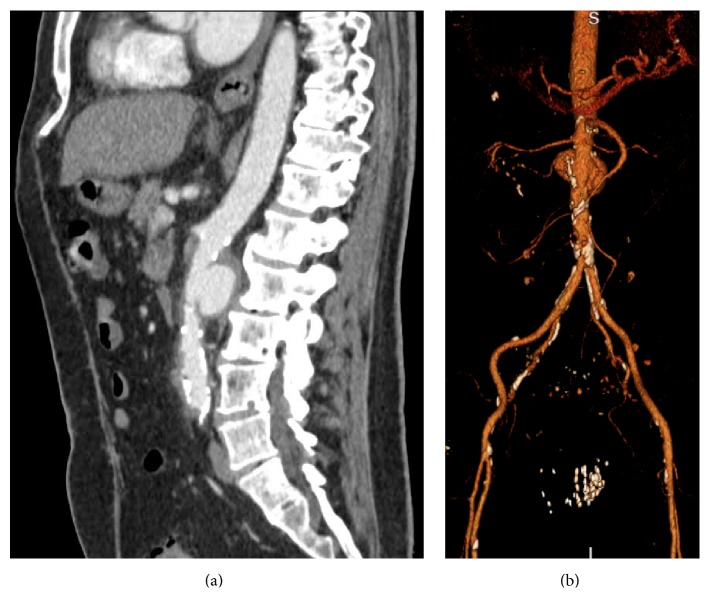
CT image and 3D reconstruction of aneurysm. (a) Sagittal CT shows saccular infrarenal aneurysm with surrounding inflammation. (b) 3D reconstruction demonstrates that the relation of the aneurysm to major abdominal arteries calcifications can be readily identified.

**Figure 2:**
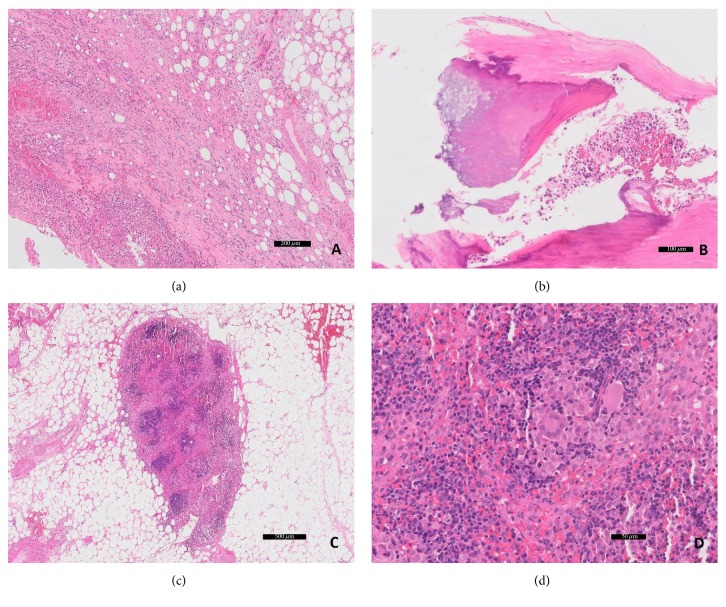
Microscopic (hematoxylin and eosin stain) photos of the resected portion of the aorta. (a) Periaortic adipose tissue with abundant chronic inflammation and fat necrosis. (b) Atherosclerosis and calcifications can be readily identified. (c) Perivascular lymph nodes show granulomatous inflammation. (d) Occasional giant cells are present in the lymph nodes.

In the midst of these recurrent hospitalizations, the patient transferred his care to a urologist with our group. He was found to have positive urine cytology which was concerning for a recurrence of his cancer and a CT urogram was ordered as follow-up (Figure 1). This CT, now 8 months from his most recent BCG treatment, was notable for two abdominal aortic aneurysms, both infrarenal. Given that these aneurysms were not present on his imaging 7 months earlier, there was a concern for a mycotic process. He was admitted and underwent open resection of his aneurysms with inline reconstruction utilizing a rifampin-soaked Gelsoft 16 × 8 mm bifurcated graft.* Mycobacterium* complex DNA was isolated from fluid around his aortic wall for which he was started on rifampin, ethambutol, and isoniazid (Figure 2). His postoperative course was complicated by chylothorax and GI bleed requiring transfer to an ICU and multiple transfusions. He was discharged after a month in good condition with a follow-up and a plan for 6 months of therapy with rifampin, ethambutol, and isoniazid.

## 3. Discussion

Intravesicular BCG is the standard-of-care adjuvant therapy for the treatment of high-risk non-muscle-invasive bladder cancer [6]. BCG has been shown to decrease the likelihood of both recurrence and progression after TURBT, as well as eradicate residual tumor in greater than 70% of patients with carcinoma in situ [1, 6, 7]. BCG is generally well tolerated, with most side effects directly related to the T-cell mediated inflammatory response that is believed to drive the antitumor effect of the treatment [6]. As such, BCG therapy can be thought of as an iatrogenic cystitis, with common symptoms including urinary frequency, dysuria, and a low grade fever lasting 24 to 48 hours.

However, around 5% of patients can experience significant morbidity, typically noncaseating granulomatous inflammation of either local structures within the genitourinary tract or systemic organs, including the lung, liver, and osteomuscular structures [8]. These reactions were initially believed to be aseptic, hypersensitivity responses given that organisms could rarely be cultured or identified with acid-fast stains of the affected tissue [9]. Improvements in technique over time and the advent of PCR have led to a revision of this position, but a 2014 review found that acid-fast staining, mycobacterial culture, and PCR-based assays were only positive in 25.3%, 40.9%, and 41.8% of cases, respectively. The authors emphasized the role of exclusion of other entities and prompt response to antituberculosis treatment in making the diagnosis of BCG infection [10].

Furthermore, the time to presentation of these symptoms can vary dramatically, from days to years after the last instillation [10]. As such, making the diagnosis can be challenging, especially given that the presenting symptoms are often difficult to distinguish from more common etiologies such as pneumonia or urinary tract infection. Moreover, it appears that BCG infection can be broadly divided into two syndromes based on time of presentation after instillation. Early BCG infection tends to present within 6 months and is usually disseminated whereas late BCG infection tends to involve the genitourinary tract [8].

Our patient's particular history is complicated by multiple presentations to medical facilities resulting in diagnoses of UTI and pneumonia and concern for malignancy in the setting of pancytopenia. While it possible that he in fact suffered from several infections and has an occult malignancy, BCG sepsis with recurrent fever, pneumonitis, and bone marrow involvement resulting in pancytopenia provides a unifying explanation for the course of his illness [11, 12]. Indeed, the patient endorsed prolonged fever and shaking chills immediately following his most recent instillation and more or less continuous symptoms from then on, suggesting ongoing active mycobacterial infection. It is likely that the patient suffered an early disseminated infection which continued progressing until his diagnosis 8 months later, consistent with the existing literature.

Mycotic aneurysm is rare even among those patients experiencing systemic complications from BCG therapy, although it does occur [5]. Overall, we were only able to find 27 cases reported in the literature to date, usually infrarenal aortic aneurysms like those in our patient [5, 13]. Bladder cancer patients are more likely to have a history of smoking and as such are also predisposed to diffuse vascular disease. This provides a portal of entry for hematogenous seeding of the vaso vasorum. Mycotic aneurysms tend to present late after BCG therapy, on average 19 months after the last instillation, with a prolonged course marked by fever, malaise, and weight loss. Prognosis is poor, with a 15.8% attributable mortality rate [10]. This case is unique in that it appears that the patient suffered an early disseminated infection with bone marrow and potentially lung involvement that went unrecognized for several months.

## 4. Conclusion

BCG infection after therapy is an uncommon but serious side effect of BCG therapy. Diagnosis is challenging as BCG infection can manifest as multiple syndromes, sometimes years after treatment. Furthermore, work-up for BCG infection is often negative. Thus, it is important for clinicians to be aware of the patient's history of BCG treatment and consider BCG infection when confronting an unexplained febrile illness, especially when granulomas are seen on histology. Additionally, patients should be aware that the initial cystitis and flu-like symptoms following BCG therapy should resolve after 48 hours and that any persistence of fever, chills, or other manifestations of sepsis is a cause for concern.
